# Comparing Taste Detection Thresholds across Individuals Following Vegan, Vegetarian, or Omnivore Diets

**DOI:** 10.3390/foods10112704

**Published:** 2021-11-05

**Authors:** Fatemeh Jalil Mozhdehi, Sashie Abeywickrema, Phil James Bremer, Mei Peng

**Affiliations:** Sensory Neuroscience Laboratory, Department of Food Science, University of Otago, P.O. Box 56, Dunedin 9054, New Zealand; fatemeh.jalilmozhdehi@postgrad.otago.ac.nz (F.J.M.); sashie.abeywickrema@postgrad.otago.ac.nz (S.A.); phil.bremer@otago.ac.nz (P.J.B.)

**Keywords:** plant-based diet, omnivorous diet, vegan diet, vegetarian diet, taste detection threshold, taste sensitivity

## Abstract

Taste perception plays an undisputed role in food choice, preference, and intake. Recent literature suggests that individual diet and taste sensitivity may have a reciprocal relationship, with evidence highlighting that specific diets can alter taste sensitivities. Commensurate with an increase in the prevalence of plant-based diets is the importance of investigating if following a vegetarian or vegan diet is associated with altered taste sensitivities. In this study, the taste detection thresholds for six compounds (i.e., *sweet*—sucrose, *salty*—sodium chloride, *sour*—citric acid, *umami*—monosodium glutamate, MSG, *bitter*—caffeine, and *metallic*—iron II sulphate heptahydrate) were measured for a total of 80 healthy, New Zealand European females aged 18–45 years old, who were categorised as 22 vegans, 23 vegetarians, and 35 omnivores. Each participant’s detection thresholds to these compounds were measured across two sessions, using an ascending Method of Limits with two-alternative-forced-choice presentations. The threshold data were analysed using both multivariate (i.e., principal component analysis) and univariate (i.e., ANCOVA) techniques to assess differences across the three types of diet. Multivariate analysis suggested that the omnivore group had distinct taste sensitivity patterns across the six compounds compared to the vegetarian or vegan group, which were characterised by relatively heightened sensitivity to *metallic* and lowered sensitivity to *sweetness*. Furthermore, the vegetarian group was shown to have a significantly lower detection threshold to bitterness (i.e., caffeine) relative to the other two groups (*p* < 0.001). While future study is required to investigate the cause–effect relationship between individual diet and taste sensitivities, the present study provides a systematic evaluation of taste sensitivities of individuals following distinct diets. This information may be valuable to future gustatory research as well as to food manufacturers.

## 1. Introduction

Taste perception plays an undisputed role in food preference, choice, and consumption [1]. Recent literature suggests that individual diet and taste sensitivity may have a reciprocal relationship, with evidence highlighting that specific diets can alter taste sensitivities [2,3,4]. In recent years, the number of people following vegetarian or vegan diets has rapidly increased, with approximately 14% of the world population now following one of these diets (e.g., [5,6,7,8,9]). Internationally, young Western females have been reported to be the most likely group to reduce animal products from their diet [10,11]. In New Zealand, approximately 10% of population were following a vegetarian diet in 2019 [12]. Commensurate with this fast-pacing global dietary shift, an intriguing question arises as to whether people who follow plant-based diets have different taste thresholds to people following an omnivore diet. An answer to this fundamental question will not only enhance our understanding of links between individual taste sensitivity and their food choice, it will also have important applied implications to food and beverages manufacturers, marketers, and nutritionists [13,14].

Among different measures available for quantifying individual taste sensitivity, the taste detection threshold (DT) is one of the most common measures in the literature [15,16,17]. Recent sensory research suggests that regular consumption of certain types of foods can have substantial impacts on an individual’s taste sensitivity [18,19]. For instance, a few empirical studies have shown that individuals who consume more fat-rich food (e.g., butter, meat, dairy) tend to have a lower sensitivity to fatty acids comparing to others [2,20,21,22]. Indeed, Heinze and Costanzo [22] outlined a strong negative correlation between fat consumption and taste sensitivity by measuring individual DTs to multiple fatty acids (i.e., oleic acid, canola oil, paraffin oil) and recording their habitual intake. In addition, a number of studies have highlighted potential links between specific diets and chemosensory sensitivities. For instance, by comparing Thai and Japanese consumers, it was found that the prolonged consumption of spicy foods with strong flavours was associated with declined taste sensitivities to all five basic tastes [23]. Additionally, in a Korean study, a significantly lower taste sensitivity to salty taste (NaCl) has been reported among individuals who adopt a high-salt diet [24]. Consistently, individuals who frequently consume sweetened beverages, such as carbonated soft drinks or fruit juice, have been reported to have a lower taste sensitivity to sweetness, compared to those who have a moderate or infrequent consumption of sweetened drinks [25]. Furthermore, a recent study suggests that the Mediterranean diet (deemed as a high-salt diet) influences the sensitivity to salty taste and cause metabolic syndrome in the long-term [18].

Vegetarian and vegan diets are defined as diets containing plant foods (e.g., grains, nuts, seeds, fruits, and vegetables) with reduced or no animal products [26,27,28]. Compared to the omnivorous diet, both vegetarian and vegan diets are rich in fibre and lower in fat and protein [8,29]. While both vegan and vegetarian diets are comprised of mostly plant-based foods, their nutritional quality and intake have been reported to be different. Specifically, protein intake among people on a vegetarian diet has been reported to be substantially lower than for people who follow either a vegan or omnivore diet [30,31,32,33]. Further, recent studies have shown that vegan and vegetarian diets could be associated with high salt intake, due to the high salt content of plant-based meat alternatives [18]. Additionally, the vegan diet appears to be associated with an increased composition of carbohydrate, poly-unsaturated fatty acids, dietary fibre, and vitamins (i.e., vitamin C, E) in comparison to the vegetarian diet [34].

Despite the global trend for adopting plant-based diets, there is surprisingly little data on its effects on sensory perception. A recent study investigating differences in bitter taste sensitivity and vegetable consumption, across vegetarians, flexitarians, and omnivores, demonstrated that vegetarian participants had a lower sensitivity to the bitter compound (PROP; 6-n-propylthiouracil) relative to followers of the other two types of diets [35]. These authors cited the need for further research to better understand the sensory-diet links observed among people following distinct diets.

Building upon the existing literature, the present study aims to test for differences in taste sensitivities across people who follow either vegetarian, vegan, or omnivore diets. The present study aims to measure taste detection thresholds to six taste qualities, including *sweet*, *salty*, *bitter*, *sour*, *umami,* and *metallic*. This information will provide valuable insights for future gustatory research, as well as for food manufacturers.

## 2. Material and Methods

### 2.1. Participants

New Zealand European females aged between 18 and 45 years from the general community of Dunedin, New Zealand were invited to participate in this study. Individuals with chronic sensory dysfunction, food allergies, being pregnant, smokers, taking regular medications, or having BMI under 18.5 kgm^−2^ were excluded from the study. During the recruitment, potential participants were asked to complete a survey, which asked for their current diet category (i.e., vegan, vegetarian, omnivore, or other), and how long they had been following the selected diet. The definition of each diet category was provided in the survey. Specifically, ‘vegetarian’ was defined as individuals who had not consumed any portion of red meat or poultry for at least the past 12 months [36,37]. ‘Vegan’ was defined as individuals who had not consumed any animal product or any food derived from animals for at least the past 12 months [37,38]. The omnivore group was defined as individuals who eat a variety of food of both plant and animal origin [39,40].

A total of 80 eligible participants were recruited into the study, including 22 participants following a vegan diet (age: 23 ± 6 years), 23 participants following a vegetarian diet (age: 22 ± 6 years), and 35 participants following an omnivore diet (age: 24 ± 7 years). Participants were instructed to refrain from food and non-water beverages for at least 10 h overnight before each session. An informed written consent was obtained from each participant prior to testing. At the completion of the study, each participant was given monetary compensation. The study was approved by the University of Otago Human Ethics Committee (Reference: 19/111).

### 2.2. Overview of the Study

The study was carried out at the Sensory Neuroscience Laboratory at the University of Otago. Threshold tests were carried out in standard sensory booths. Each participant attended two 1 h morning sessions on consecutive weekdays. Each session included a threshold test for six taste stimuli, with randomised stimuli orders across sessions and participants. In total, each participant performed two replicates of the threshold test for each compound. 

### 2.3. Stimuli

A total of six taste compounds were tested in the present study, including the five basic tastes and *metallic*. The inclusion of *metallic* was due to previous data suggesting its close relevance to meat consumption [41,42]. Information on these compounds are presented in Table 1.

To enable detection threshold testing, each compound was made into a 6-step concentration serry of solutions. Preliminary in-house tests were carried out to determine the suitable concentration range for each compound. All testing solutions were carried out using a serial dilution technique, using carbon-filtered water (0.5 micron) as the solvent. After solution preparation, 5 mL of the solution was sub-sampled into a 50 mL glass tube and labelled with a 3-digit code. All samples were prepared one day before testing and kept refrigerated. On the testing day, all samples were taken out two hours before the test and calibrated to room temperature (19–20 °C) before serving.

### 2.4. Detection Threshold Tests

Each session comprised six detection DT tests. Testing followed the modified American Society for Testing and Materials (ASTM E679) method [43,44,45]. Testing orders of the taste stimuli were randomised across sessions and participants. At the start of each test, each participant was presented with a reference sample of each taste at a supra-threshold level and asked whether they could perceive a taste. This step was included to ensure that the participant was not ageusic to the testing compound. After a short break and palate cleansing, the participant was asked to perform the DT test for the corresponding taste stimulus, using the ascending Method of Limits, with a 2-alternative-forced-choice (2AFC) presentation. Notably, the samples included in the DT tests were at much lower concentrations than the reference sample in the screening test. Specifically, each DT test contained 6 pairs of 2AFC task, with each comprised a testing sample and a blank sample (containing the solvent only). Across the pairs, the concentration of the target sample followed an ascending order. In each 2AFC task, the participant was asked to indicate which one of these samples contained a taste stimulus (i.e., the sample that is not the blank). The participant was forced to produce an answer even if they were unsure. To cleanse their palate, participants were instructed to rinse their mouths with carbon-filtered water (0.5 micron) after each sample and to expectorate the rinse water. The order of these taste stimuli was counterbalanced throughout the test. A 1-min inter-trial interval was enforced, with a 10-min break between the taste stimuli. 

### 2.5. Data Analysis

#### 2.5.1. Detection Threshold Estimation

The DT was estimated following the protocol of ASTM E679. The individual’s DT was obtained by calculating the geometric mean of the last missed concentration and the next higher concentration detected, considering it as the first of all subsequent concentration correctly answered. When the DT was above or below the used concentration range, a hypothetical concentration step was calculated by dividing/multiplying the concentration at the lowest/highest step by the dilution factor. In summary, if the participant had a missed detection for the lowest tested concentration, the threshold was taken as the geometric mean of the lowest concentration presented in the series and the hypothetical one below it. Conversely, if the participant had a missed detection for the highest tested concentration, the threshold was taken as the geometric mean of the highest concentration in the series and the hypothetical one above it.

#### 2.5.2. Statistical Analysis

A series of univariate analyses were carried out to test differences in the Detection Threshold (DT) across the three diet groups (i.e., vegan, vegetarian, omnivore) for each taste stimuli. During the analyses, age was treated as a continuous covariate. Post hoc tests with Benjamini–Hochberg (BH) corrections were used to explain any significant effect, which was detected at *p* < 0.05.

Subsequently, separate principal component analyses (PCA) using a correlation matrix were performed to determine relationships among the DTs of six taste stimuli for each of the vegan, vegetarian, and omnivore diet groups. An additional PCA was employed to assess taste sensitivity patterns across the vegan, vegetarian, and omnivore groups. PCA bi-plots were implemented to present the results. Any visually identifiable relationships were discussed. All of the statistical analyses were performed using R software (Version 1.1.463, R, RStudio Public Benefit Corporation, Boston, MA, USA). 

## 3. Results

### 3.1. Participant Characteristics 

The present study included 80 New Zealand European females comprised of 23 vegans, 22 vegetarians, and 35 omnivores. The age of participants ranged between 18 and 44 years (mean age = 23 ± 6 years) within the vegan group, 18 and 33 years (mean age = 22 ± 4 years) within the vegetarian group, and 18 and 44 years (mean age = 24 ± 7 years) within the omnivore group. 

### 3.2. Comparing Taste Sensitivity Patterns across the Vegan, Vegetarian, and Omnivore Groups

Table 2 reports the mean values of DT to each of the testing compounds derived from the separate groups. All values fall within the concentration range used for each compound. Notably, the mean DT of the vegan group to the sweet taste was relatively close to the lower end of the concentration range (0.113 g·L^−1^). Closer inspections of individual data did not reveal any abnormality with DT calculations for this group. 

A series of univariate analyses were employed to test the differences in the DT across the three diet groups (i.e., vegan, vegetarian, omnivore) for each taste stimuli. The univariate analysis employed on the *bitter* taste data revealed a significant main effect from the diet group. The post hoc tests with BH corrections suggested that the vegetarians had a significantly lower DT compared to both vegans and omnivores (all *p* < 0.001). No significant difference was observed between vegan and omnivores; see Table 2. In addition, the separate univariate analyses suggested that diet group had no main effect over *sweet*, *salt*, *sour*, *metallic*, or *umami* taste detection thresholds. 

**Figure 1:**
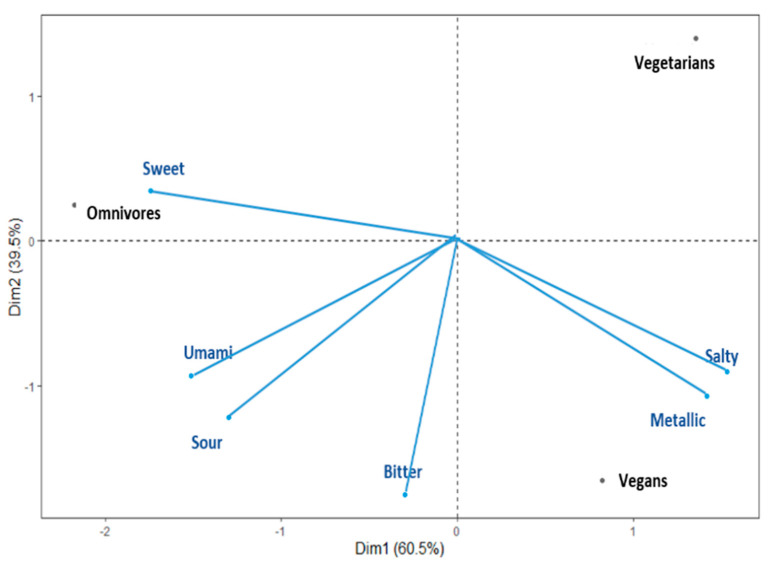
Principal component bi-plot graph for the vegan, vegetarian, and omnivore groups.

A PCA using a correlation matrix was performed to determine relationships among DTs of the six taste stimuli and three diet groups. Diet groups (i.e., vegan, vegetarian, omnivore) and DT variables were merged in a single bi-plot graph to further facilitate visualisation (see Figure 1). The two main principal factors (F1 and F2) of the bi-plot explained 100% of the total variability and depicted that the DTs for *sweet*, *salty*, and *metallic* tastes were the most discriminating variables across the diet groups. The vegan group was positioned on the overall PCA similarly to the vegetarian group with regard to PC1. Specifically, the bi-plot suggests that the omnivores group was dominated by the sweet DT, and vegans and vegetarians were dominated by the *salty* and *metallic* DTs. Additionally, the vegetarian group showed lower domination from the *bitter*, *sour,* and *umami* DTs. 

### 3.3. Profiling Taste Sensitivities for Vegan, Vegetarian, and Omnivore Groups

**Figure 2:**
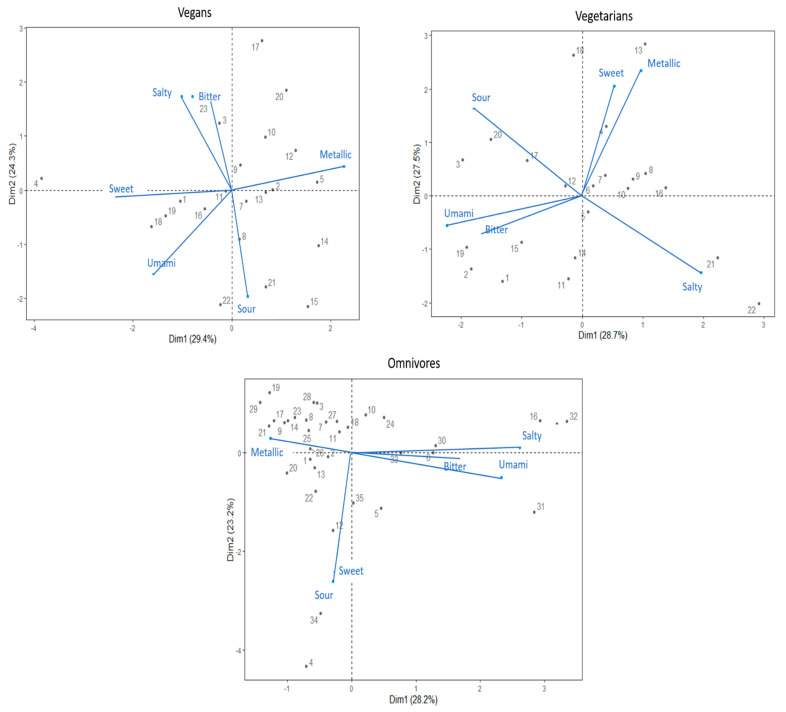
Bi-plots based on principal component analysis based on detection thresholds to six taste compounds. The plots represent data derived from the vegan, vegetarian, and omnivore group, respectively. Numbered points of the PCA represent individual participants by their ID number.

Separate PCAs were used to determine relationships among DTs of the six taste stimuli in each diet groups. The DT variables of each diet group were merged in separate bi-plot graphs to further facilitate the visualisation (see, Figure 2). 

The first two main principal components (PC1, PC2) of the vegans PCA explained 53.7% of the variance. The PCA bi-plot suggested that *salty* and *bitter* DT variables show a high positive correlation, while both *sweet* and *salty* DTs were negatively correlated with the *sour* DT. Additionally, *umami* DT indicated a positive correlation with *sweet* and *sour* DTs. Both *sweet* and *umami* DTs showed negative correlation with the *metallic* taste. 

The PC1 and PC2 of PCA of vegetarians explained 56.2% of the variance. Both the *sweet–metallic* and *umami–bitter* taste DTs variables showed high positive correlations. Furthermore, *sour* and *salty* DTs showed a negative correlation. 

The first two components (PC1, PC2) of the omnivores PCA explained 51.4% of the variance. PCA depicts that *sweet* and *sour* taste DTs have a high positive correlation. Additionally, *bitter*, *salty,* and *umami* DTs showed high positive correlation among variables, with a negative correlation with the *metallic* DT. Specifically, the omnivores’ PCA bi-plot suggests that the majority of the group is dominated by the *metallic* DT. 

## 4. Discussion 

The present study provides the first systematic evaluation of taste detection thresholds among individuals following either a vegan, vegetarian, or omnivore diet. The results indicated that people following a vegetarian diet had a significantly lower DT to bitter taste (i.e., a higher sensitivity) compared to people on either vegan or omnivore diets. Furthermore, the omnivore dietary group, relative to the other two groups, had a higher DT in sweetness. By contrast, the vegan group had a relatively higher DTs for the *salty* and *metallic* compounds.

The multivariate sensory data in the present study suggested that the omnivore group showed a distinct sensory sensitivity pattern across the six compounds compared to the vegan and vegetarian groups. Notably, the omnivores group differed considerably from the other two groups on the PC that was primarily driven by *metallic* and *sweet*. Relatively, individuals on an omnivore diet were more sensitive to *metallic taste* but less sensitive to *sweetness*. This observation with regard to *metallic* is in direct contrast to earlier studies that have proposed that the frequent consumption of red meats may reduce sensitivity to *metallic* taste [41,42,46]. The difference between the present study and that of Ambaldhage and Puttabuddi [46] may be related to the use of different sensory measures. This earlier study measured supra-threshold intensity perception, whereas the present study measured detection thresholds. Furthermore, inconsistencies in findings may suggest that long-term meat consumption may differentially alter low- and high-order sensory processing. Notably, previous research has also indicated that sensitivity to the *metallic* taste could be linked to consumption of artificial sweeteners, with increased sensitivity linked to higher consumption [47,48]. However, this link was not directly assessed in this study.

With regard to differences in *sweetness* perception across the diet groups, there appears to be little comparable data among human participants. However, rodent studies have reported that frequent and long-term consumption of a fat-rich diet can be associated with enhanced sensitivities to fatty acids and relatively reduced sensitivities to other taste qualities (e.g., [49,50,51]). Liu and Walter [52] have also reported a close link between *sweetness* sensitivity and the tendency of adopting an omnivore diet in several species, with this specific diet associated with increased sensitivity. These authors proposed that these phenotypes may be linked to the selective constrains of *TAS1Rs* genes.

In this study, *bitterness* (i.e., caffeine) was the only compound that led to significant differences in sensitivity across the groups on different diets. These results can be contrasted to a recent study by Cliceri and Spinelli [35], who specifically tested for differences in intensity perception of bitterness (using 6-n-propylthiouracil; PROP) across omnivores, flexitarians, and vegetarians. Assessments of bitterness perception across vegetarian and other diets were motivated by the assumption that bitterness perception is linked to fruit and vegetable consumption. While Cliceri and Spinelli [35] did not obtain sensitivity measures, their results implied that individuals following a vegetarian diet were less sensitive to the bitter taste by perceiving the PROP compound to be weaker in intensity. However, our results directly contradict their findings. Specifically, vegetarian participants in the current study showed significantly lower DT (i.e., higher bitter sensitivity) than others. These differences may be explained by the different compounds used to elicit bitterness. While the present study tested bitterness perception with caffeine, Cliceri and Spinelli [35] used PROP. However, the differential results were not surprising given the well-documented phenomenon of compound-specific transduction for bitterness (e.g., [51,53]). Notably, the vegan and vegetarian groups yielded similar PCA, particularly with regard to PC1, despite clear difference relating to *bitterness*. 

The present study had some limitations. First, classifications of the diet groups were based on self-reported measures, as opposed to empirical data on food consumption. Potentially, tracking the consumption frequency of specific foods can explain additional inter-individual variability in gustatory sensitivities. For instance, sensitivity to metallic may be related to the high consumption of fish due to the presence of heavy metal elements. Second, this study included a relatively small sample size compared to previous studies on taste detection thresholds [54,55,56]. Additionally, only healthy young females were tested. The small sample size with a highly homogenous group of participants should be considered a caveat of the study.

Overall, the present study systematically evaluated the differences in taste sensitivities across people who follow either a vegetarian, vegan, or omnivore diet. With multivariate sensory data, some clear differences were shown in the sensory sensitivity patterns between the omnivore versus the non-meat dieters, with the former group being characterised by relatively heightened sensitivity to *metallic* and a lowered sensitivity to *sweetness*. While the vegan and vegetarian groups had relatively close sensory sensitivity profiles, the vegetarian group showed a significantly lower sensitivity to bitterness. While future studies are required to replicate the findings in other sub-populations with various demographic parameters, the current study generates important and timely insights into links between vegetarian/vegan diets and taste sensitivities. This information will be of value to future gustatory research as well as to food manufacturers.

## Figures and Tables

**Table 1:** Information of the taste compounds and their concentration range used in the present study. The specific concentration steps between the lowest and highest concentrations can be calculated by multiplying the lowest concentration by the dilution factor.

Taste	Prototypic Compound	Supplier	Purity	Concentration Range (g·L^−1^)		Dilution Factor
				Lowest	Highest	
Sweet	Sucrose	Chelsea, New Zealand	97%	1.13 × 10^−1^	8.00	2.34
Bitter	Caffeine	Simple Nutritions, Turkey	98%	6.40 × 10^−5^	0.20	5.00
Salty	Sodium Chloride	Cerebos, New Zealand	96%	1.20 × 10^−3^	1.30	4.30
Metallic	Iron II Sulphate Heptahydrate	PipingRock, England	99%	4.00 × 10^−5^	0.04	4.00
Umami	Monosodium Glutamate (MSG)	Miwon, Korea	99%	9.20 × 10^−2^	0.70	1.50
Sour	Citric Acid	Hansells, New Zealand	98%	5.00 × 10^−2^	1.00	1.80

**Table 2:** Detection thresholds (means and standard deviation in g·L^−1^) of participants in three diet groups for the six taste stimuli used. Within a row, means with different letters are statistically different at *p* < 0.05. Significant main effects (*p* < 0.05) from the diet groups are indicated by asterisks.

Taste Stimuli	Vegans	Vegetarians	Omnivores	F Statistic	*p* Value
Sweet	1.205 ± 2.08 A	1.63 ± 2.15 A	2.33 ± 2.49 A	1.94	0.151
Salty	0.210 ± 0.47 A	0.211 ± 0.62 A	0.182 ± 0.44 A	0.02	0.979
Bitter	0.107 ± 0.16 A	0.049 ± 0.13 B	0.085 ± 0.16 A	8.37	<0.001 *
Sour	0.101 ± 0.11 A	0.084 ± 0.04 A	0.101 ± 0.02 A	0.86	0.429
Metallic	0.025 ± 0.01 A	0.022 ± 0.01 A	0.016 ± 0.01 A	1.04	0.359
Umami	0.230 ± 0.13 A	0.205 ± 0.14 A	0.237 ± 0.22 A	0.32	0.726

## Data Availability

Data has not been deposited in any repository, however it can be made available to researchers upon request.
